# Quantitative approach to numbers and sizes: Generation of primary neurospheres from the dorsal lateral ganglionic eminence of late embryonic mice

**DOI:** 10.12688/f1000research.21208.2

**Published:** 2020-03-11

**Authors:** Christopher Blackwood

**Affiliations:** 1 Department of Biomedical Sciences, Cornell University, Ithaca, NY, 14853, USA

**Keywords:** Embryonic, mechanical dissociation, neurosphere, neural stem cell, progenitor cell

## Abstract

**Background:** The neurosphere assay is a powerful *in vitro* tool to investigate neural stem cells in the dorsal lateral ventricle (dLGE). In the dLGE, metrics of sizes and numbers of neurospheres generated using this assay has not been completely characterized. The objective of this protocol is to provide a stepwise method from a single isolation that predicts the average number of neurospheres generated and to estimate an approximation of its sizes after several days *in vitro*. The advantage of this protocol is that no expensive and specialized equipment is needed for tissue isolation. Estimates about the numbers and sizes of neurospheres will provide investigators with quantitative data to advise on how much starting dLGE tissue is required to generate the appropriate number of spheres for the implementation of downstream applications, including immunocytochemistry, self-renewal and differentiation assays.

**Methods:** Our method is based on a simple dissection technique, where tissue surrounding the dorsal lateral ventricle from a single mouse embryo is trimmed away to enrich for neural stem cell and progenitor populations. Following this dissection, tissue is mechanically dissociated by trituration. Cells are then cultured in media containing epidermal growth factor and other supplements to generate healthy primary neurospheres.

**Results:** Using this approach, we found reproducible number of primary neurospheres after 7 days *in vitro *(DIV). Furthermore, we observed that this method yields an average range of neurospheres sizes greater than 50 μm, but less than 100 μm after 7 DIV. Lastly, using an anti-GFAP antibody, we show that these neurospheres can be stained, confirming their use in future immunocytochemistry studies.

**Conclusions:** Future use of this protocol provides metrics on the generation of primary neurospheres that will be useful for further advances in the area of stem cell biology.

## Introduction

Neural stem cells are a tissue-specific subtype of self-renewing and multipotent cells that will produce several mature cell types. The neurosphere assay is an important tool that has been extensively employed to study neural stem cell biology ^1^. Since its introduction some 25 years ago ^2^, neurospheres have been used to study neurogenesis ^3^, genes that regulate self-renewal ^4, 5^, and molecular mechanisms that control neuronal and glial differentiation ^3, 6– 9^. Although there are many neurosphere protocols, the expected number and size of neurospheres generated after a week *in vitro* is not entirely characterized.

We developed a simple dissection technique that helps to maximize the number of neurospheres that can be produced in culture. Furthermore, we characterize the expected sizes of neurospheres after a week *in vitro*. With some other techniques, a brain slicer or other means are used to obtain thick slices of brain tissue from late embryonic stages ^10, 11^. The area surrounding the ventricle is then microdissected from a given slice of tissue to enrich for neural stem/progenitor cells. This approach, while effective, can be painstaking and may require expensive specialized equipment. Additionally, many protocols do not provide metrics on expected numbers and sizes of neurospheres generated. Thus, it is unclear whether researchers can generate sufficient numbers of neurospheres in a particular range of sizes. In contrast, our approach requires no specialized equipment. The lateral ventricle is visualized with a stereomicroscope, and the surrounding tissue is simply trimmed away using a razor blade or scalpel. This method requires only half of a single brain, and generates reproducible numbers of neurospheres in a few days. Furthermore, using our method neurospheres small appears as early as 3 days. Another advantage of this protocol is that it can generate neurospheres with average sizes of 50 μm - 100 μm after 5–7 days *in vitro*.

## Methods

### Mice

The animals were housed in the AAALAC-accredited East Campus Research Facility and Transgenic Mouse Core Facility in the Veterinary College of Cornell University. All animal procedures were performed in accordance with the guidelines outlined in the National Institutes of Health (NIH) Guide for the Care and Use of Laboratory Animals, eighth Edition. Animals were approved by Cornell University’s Animal Care and Use Committee (IACUC; #01-75). Mice were maintained on a mixed 129Sv/C57BL/6 background and housed on a reverse light-dark cycle. Food and water were continuously available. Male and female mice mated overnight. The following morning females were separated and checked for vaginal plug. Pregnant mice were euthanized using CO _2_ asphyxiation followed by cervical dislocation consistent with the recommendations of the Panel on Euthanasia of the American Veterinary Medical Association and the Cornell IACUC. Embryonic day 17.5 (E17.5; E0.5 was defined as the first detection of the vaginal plug) male and female embryos were dissected. A total number of n=5 mice were used for primary neurospheres experiments.

### Collection of tissue

**Figure 1.**
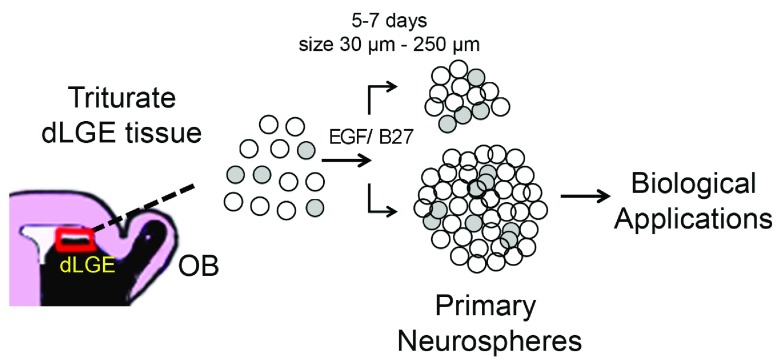
Schemetic illustration. Tissue from the dLGE is dissected and dissociated. Single cells are grown in media containing EGF and B27. After 7 days *in vitro* neurospheres are on average between 50 μm to 100 μm in size. Neurospheres generated can be used for various downstream applications.

The mouse brain was sliced down the center into two hemispheres. Using one hemisphere, placed sagittal orientation, dissect out the dorsal lateral ventricle (see Figure 1). The dissections were guided using stereotaxic coordinates (A/P 1 mm, M/L 1 mm, D/V 2.3 mm) from Paxinos and Franklin (2007) atlas source.

### Immunofluorescence

Neurospheres were fixed with 4% paraformaldehyde, and blocked in goat serum containing 0.5% Triton. Immunofluorescence analysis of protein expression was performed using rabbit anti-Glial Fibrillary Acidic Protein (GFAP) Antibody (Millipore; RRID:AB_2109645; ab5804; 1:100). Secondary antibodies used were biotinylated goat anti-rabbit (Abcam; RRID:AB_2661852; ab64256; 1:1000) and streptavidin alexa fluor 488 conjugate (ThermoFisher Scientific; RRID:AB_2315383; S11223; 1:500).

### Data acquisition and statistics

Images were taken with a Canon EOS Rebel XS camera. (Canon USA; Melville, NY). The optimum magnification is approximately 5x with 3888 x 2592 dimensions. Camera was connected to the trinocular port of the stereomicroscope (Carl Zeiss Stemi 305; White Plains, NY) using Mount Adaptor EF-EOS (6098B007AA; Canon; Melville, NY). The working distance was defined as the amount of room required between the top of the neurosphere and the bottom of the objective lens in order for the image to be in focus. The steromicroscope was used at a working distance of ~110 mm. Due to the variation in neurosphere size, 110 mm should be adjusted to focus on the desire region of the neurosphere to provide optimal focus. The field of view represents a length of 783 μm and a width of 522 μm. Data is derived from single random pictures of each well. Per animal, 3–4 wells were analyzed. A total of 5 individual animals were analyzed. Size measurements and neurosphere counts were analyzed in Adobe Photoshop (version 10.0.1) CS3 Extended (Adobe; San Jose, CA) using the measurement function (ImageJ is an open-access alternative that can be used to perform this function). Statistical analyses were carried out as previously described ^12, 13^. Briefly, data were analyzed using PRISM 8 (version 8.3.0) (GraphPad Software; San Diego, CA) by performing one-way ANOVA. If the main effect was significant (p < 0.05), Bonferroni’s multiple comparison post hoc test were used to compare the different replicates. The null hypothesis was rejected at p < 0.05. Data is made available on figshare open access platform (Metrics of Primary Neurospheres). Error bars represent standard error of the mean (±SEM).

### Neurosphere assay protocol

This protocol is designed to generate neurospheres from a single embryo. Multiply all values as needed to generate neurospheres from additional embryos. See Table 1 and Table 2 for premade solutions and materials needed.

**Table 1.** Equipment, reagents, and catalog information.

Name of material/equipment	Type	Company	Catalog Number
Industrial Razor Blades	Surgical tool	VWR	55411-050
Forceps	Surgical tool	Fine Science Tools	11251-20
Small Scissors	Surgical tool	Fine Science Tools	14060-09
Hanks' Balanced Salt Solution (Adjust to pH 7.1 after dilution to 1X)	Reagent	ThermoFisher Scientific	14185-052
0.25% Trypsin/EDTA (1x)	Reagent	ThermoFisher Scientific	25200-056
MgSO _4_	Reagent	JT Baker	2500-01
DNase I	Reagent	Roche	10104159001
BSA	Reagent	Sigma	A3912
10% FBS	Reagent	ThermoFisher Scientific	26400044
Penstrep	Reagent	ThermoFisher Scientific	15140-148
Soybean Trypsin Inhibitor	Reagent	Sigma-Aldrich	T6522
B27 Supplement	Reagent	ThermoFisher Scientific	17504-044
EGF Recombinant Human Epidermal Growth Factor	Reagent	ThermoFisher Scientific	PHG0311
18-gauge Needle	Dissociation tool	Becton Dickinson	305196
21-gauge Needle	Dissociation tool	Becton Dickinson	305190
23-gauge Needle	Dissociation tool	Becton Dickinson	305194
Syringes	Dissociation tool	Becton Dickinson	309657
15 ml Centrifuge Tube	Culture ware	Corning	430791
100 mm Petri Dish	Culture ware	ThermoFisher Scientific	150466
35 mm Petri Dish	Culture ware	ThermoFisher Scientific	150460
48 Well Plate	Culture ware	Corning	3548
EOS Revel Camera model # 1894C002	Imaging	Canon	3548
Digital Incubator, model #311D	Incubator	The lab Depot	15311-D

**Table 2.** Premade solutions for neurosphere assay.

Solutions	Ingredients
DMEM/F12 Serum containing media	DMEM/F12 media with 10% FBS, 1X Penstrep
Hank's-low	1x Hank's buffer with 1.2 mM MgSO4, 40 mg/ml DNaseI, 3 mg/ml BSA, and filter sterilized
Hank's-high	1x Hank's buffer with 1.2 mM MgSO4, 40 mg/ml DNaseI, 4% BSA, and filter sterilized
Neurosphere Media	DMEM/F12 media with 1x B27 and 10ng/ml EGF
Trypsin Inhibitor solution	DMEM/F12 with 1 mg/ml soybean inhibitor

**1. Set-up prior to tissue dissection**

NOTE: *Breeding and euthanasia of all animals should be performed in accordance with an institutionally approved animal care and use protocol. Sterilize all surgical instruments packed in aluminum foil in an autoclave at 121°C (15 psi) for 30 mins. This includes a scissors, forceps, and razor blades. Before starting all premade solutions should be warmed to 37°C.*

1.1) Establish breeding pairs of mice to obtain embryonic day 17 (E17) embryos. Day 0 is defined as the day a vaginal plug is detected.

1.2) Prepare sterile surgical tools (scissors for decapitation, #5 forceps, razor blades).

1.3) Add 20 mls of Hank’s buffer to each of two 10 cm petri plates and place on ice. Add 5 mls Hank’s buffer to a 15 ml tube and also place on ice. Reserve another 50 mls of room temperature Hank’s buffer.

1.4) Prewarm 10 mls of Hank’s-low BSA at 37°C

1.5) Prewarm 5 mls of Hank’s-high BSA at 37°C.

1.6) Prewarm 10 mls of DMEM/F12 with serum at 37°C.

1.7) Prewarm 5–15 mls of neurosphere media at 37°C. Amount is based on number of desired wells.

1.8) Prewarm 2 mls of 0.25% trypsin/EDTA at 37°C.

**2. Tissue dissection**

NOTE: *Make freshly prepared 70% ethanol spray prepared. Have a petri dish (100 mm) prepared with ice-cold Hank’s buffer kept on ice, which will be used to collect embryos after dissection. Afterwards, additional petri dishes will be needed to place in each of the dissected brains (35 mm)*.

2.1) Spray the abdomen with 70% ethanol, and make an incision to expose the uterus. Remove the uterus and transfer it to an empty petri plate.

2.2) Remove embryos from the uterus, spray desired number with 70% ethanol, and decapitate one or more embryos. Rinse each decapitated head in one petri plate containing ice-cold Hank’s buffer, and then transfer to the second petri plate containing Hank’s buffer on ice.

2.3) Use forceps to remove the skin and skull. Remove the brain and place in an empty petri dish.

2.4) Separate the two hemispheres with a razor blade, and place one half of a brain on its lateral surface.

2.5) Using a stereomicroscope, identify the location of the lateral ventricle on the medial surface (the dorsal region of the lateral ventricle contains the dLGE). The ventricle is visible as a T-shaped structure that is slightly darker than the rest of the brain. Using a razor blade or a scalpel, sequentially trim away the brain surrounding the ventricle on all four sides.

2.6) Transfer the dissected tissue into the 15 ml tube of Hank’s buffer on ice.

2.7) If neurospheres are to be isolated from additional embryos (e.g. because of low yield), keep tube on ice until all dissections are complete.

**3. Primary neurosphere culture**

*NOTE: Before starting warmed to 37°C the following solutions: trypsin/EDTA serum media, Hank’s-low, and Hank’s-high. In this section you will need the 18-gauge, 21-gauge, 23-gauge needle will be needed for trituration steps. Trituration should be performed gently and slowly to avoid killing cells. Hemocytometer will be needed to count cells*.

3.1) Spin sample at 300 RCF in a clinical centrifuge for 3 min. to pellet tissue.

3.2) Aspirate off the supernatant and add 2 mls of pre-warmed trypsin/EDTA. Incubate at 37°C for 15 min. with intermittent swirling.

3.3) Spin tube at 300 RCF for 2 min.

3.4) Add 10 mls of room temperature Hank’s to trypsin/tissue mixture and incubate at 37°C for 5 min. with intermittent swirling. Spin culture at 300 RCF for 3 min. and remove the supernatant.

3.5) Repeat wash step 3.4 two additional times.

3.6) Aspirate the supernatant and add 4 mls of Hank’s-low BSA.

3.7) Triturate the tissue gently and slowly approximately 10 times with an 18-gauge needle until tissue chunks appear relatively uniform in size. Avoid creating bubbles or foam.

3.8) Triturate the crude cell suspension gently and slowly approximately 7–10 times with a 21-gauge needle until tissue chunks appear relatively uniform in size.

3.9) Triturate the suspension approximately 4–5 times with a 23-gauge needle until suspension appears uniform.

3.10) Add 3 mls of Hank’s-high BSA solution to a 15 ml tube. Slowly add the cell suspension to the bottom of the tube underneath the Hank’s-high BSA solution using a 23-gauge needle.

3.11) Centrifuge at 300 RCF for 5 min.

3.12) Aspirate supernatant and resuspend cells with 3 mls of prewarmed Hank’s-low BSA.

3.13) Centrifuge at 300 RCF for 5 min.

3.14) Aspirate supernatant, and resuspend cells in 5 mls of prewarmed DMEM/F12 with serum.

3.15) Incubate tubes for 2–4 hours at 37°C to reduce bacterial contamination.

3.16) Centrifuge at 300 RCF for 5 min.

3.17) Resuspend cells in 1 ml of prewarmed neurosphere media.

3.18) Count cells with a hemocytometer. Plate 10,000 cells in a volume of 250 μl in each well of a 48-well plate. Plate at least 8 wells to ensure adequate numbers of neurospheres.

3.19) Incubate at 37°C in a humidified incubator with 5% CO _2_.

3.20) Neurospheres should form within 3–4 days. At day 3, add an additional 100 μl of neurosphere media to each well.

### Secondary neurospheres

The extended version of our protocol can be used to obtain secondary neurospheres ( https://dx.doi.org/10.17504/protocols.io.823hygn).

## Results

### This approach generates consistent number of primary neurospheres

**Figure 2.**
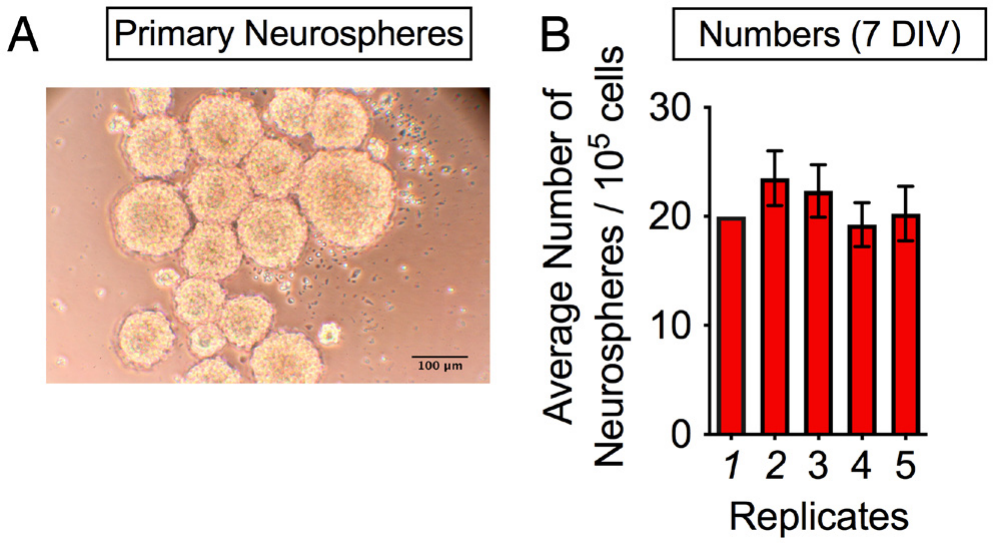
Primary neurospheres generated from the dorsal lateral ventricle. ( **A**) Average size of primary neurospheres per field of view after 7 days *in vitro*. ( **B**) Average number of neurospheres per field of view after 7 days *in vitro* (N=5). Scale bar = 100 μm.

Figure 1 shows a general overview of the neurospheres assay. A picture representation of primary neurospheres grown for 7 days *in vitro* is given in Figure 2A. The statistical analysis using a one-way ANOVA revealed no significant difference between the average numbers of neurosphere per field of view (F _(4,13)_ = 0.666; p = 0.6268; N=5) ( Figure 2B).

### This protocol generates different sizes of neurospheres

**Figure 3.**
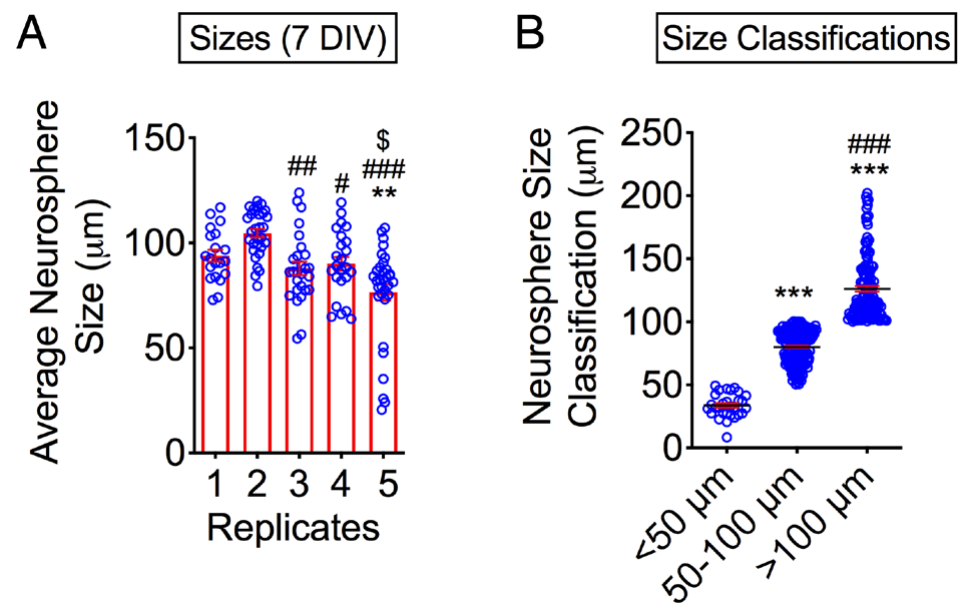
Size classification of primary neurospheres from the dorsal lateral ventricle. ( **A**) Average size of primary neurospheres per field of view after 7 days *in vitro* (N=5). ( **B**) The comparison of the numbers of neurosphere that are less than 50 μm, between 50-100 μm, and greater than 100 μm. Key to statistics **, *** = p <0.01, 0.001, respectively, in comparison to NS less than 50 μm or replicate 1. #, ##, ###, = p < 0.05, 0.01, 0.001, respectively, in comparison to NS between 50-100 μm or replicate 2. $ = p <0.05 in comparison to replicate 3 (minimum of 5 independent samples; N=29, N=214, N=136, respectively to NS<50 μm, NS between 50 μm -100μm, NS>100 μm).

Figure 3 shows in the variation in sizes of neurospheres grown for 7 days *in vitro* ( Figure 3A). The statistical analysis using one-way ANOVA revealed a significant difference in the sizes of neurospheres between the replicates (F _(4,129)_ = 11.666; p < 0.0001) ( Figure 3A). Using similar analyses, we found significant differences between the size classification of neurospheres that were less than 50 μm, between 50–100 μm, and greater than 100 μm (F _(2,379)_ = 424; p < 0.0001) ( Figure 3B). Post hoc analysis using Bonferroni’s multiple comparison revealed a significant difference between the primary neurospheres that were greater than 100 μm compared to neurospheres that were less than 50 μm (p <0.0001) or between 50–100 μm (p <0.0001) ( Figure 3B). Similarly, we found a substantial difference between primary neurospheres that were greater than 100 μm compared to neurospheres that were between 50–100 μm (p <0.0001) ( Figure 3B). Numbers and sizes of neurospheres, alongside the raw images used to produce these values, are available as *Underlying data* ^14^.

### Primary neurospheres at 7 days *in vitro* can be used for immunocytochemistry

**Figure 4.**
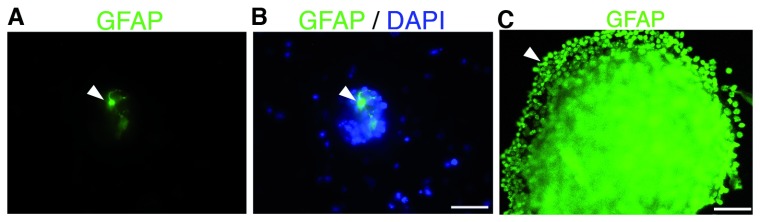
Immunocytochemistry of small and large primary neurospheres. Visual representation of immunocytochemistry staining of a small neurosphere using ( **A**) anti-GFAP antibody (green) and ( **B**) counterstained with DAPI (blue) after 7 days *in vitro* (7 DIV) Scale bar = 100 μm. ( **C**) Anti-GFAP staining of a large neurosphere 7 DIV. Scale bar = 50 μm Arrowheads represent positive GFAP signal.

Neurospheres can be used for a variety of purposes, including immunocytochemistry. Figure 4 is a picture of a small ( Figure 4A-B; arrowhead) and a larger ( Figure 4C; arrowhead) primary neurosphere immunostained using an anti-GFAP antibody and counterstained with DAPI (Figure B; arrowhead). Additional neurosphere staining using other antibodies can be found in previous published studies ^3^ from our lab.

## Discussion

There are several key steps that are important to maximize the yield and health of neurospheres. The most important step is to incubate the final triturated culture in the prewarmed DMEM/F12 with serum for 2–4 hours. This incubation time is necessary in order for the antibiotics in the media to inhibit growth of bacteria. A sign of bacteria contamination is reduced visibility of the media. Another marker of an unhealthy culture is a large number of differentiated neurons surrounding neurospheres. Indicators of differentiation are the large presence of axons and dendrites in your cultures. This can be caused by depletion of growth factors. If this is the case, it is recommend that you increase the concentration of EGF. Another cause of differentiation is too many cells in your prep. This leads to over crowdedness. It is recommend to split the culture to a lower density or decrease the number of neurons that are plated per 48 well.

Another essential step is to perform the trituration as gently as possible. Over-trituration, or trituration with great force, will result in increased cell death. After trituration, if a uniform suspension has not been achieved an alternative method used in previous protocols are strainers ^15^.

Additional modifications to the approach may be required. We have provided general guidelines for the number of cells to be plated per well and the expected number of neurospheres per field of view. If too many cells are plated per well, differentiation may occur. If so, either reduce the number of cells plated per well or increase the concentration of EGF to 20 ng/ml. If desired, trypsin inhibitor can be substituted for the FBS during the generation of primary neurospheres to inactivate proteolytic activity.

Our approach utilizes a mechanical trituration to dissociate cells. By sequentially processing the cells through successively higher gauge needles, we obtain very few clumps of cells. This eliminates the need for a cell strainer, which can reduce yield, as well as the time needed to perform enzymatic dissociation. Although cell death can be increased with mechanical dissociation, in our hands, this does not seem to affect neurosphere yield or health.

One advantage of our approach is the ease with which tissue surrounding the lateral ventricle can be isolated from the rest of the brain. Although this dissection is relatively crude, it is easier and faster than other approaches, which may require a brain slicer and/or microdissection ^10, 11^. This method eliminates the need for specialized equipment while generating high numbers of neurospheres from a single brain. This method has been shown in other studies to generate neurospheres greater than 300 μm in size when grown for 12 days *in vitro* ^3^.

## Data availability

### Underlying data

Figshare: Metrics of Primary Neurospheres.xlsx. https://doi.org/10.6084/m9.figshare.10280288.v1 ^14^.

This project contains the following underlying data:

- Spreadsheet containing numbers and sizes per field of view.xlsx (details on numbers and sizes of neurospheres produced from each mouse).
- All raw, unprocessed microscope images used to produce results.

Data are available under the terms of the Creative Commons Attribution 4.0 International license (CC-BY 4.0).
