# Supplementary material for: Quantitative approach to numbers and sizes: Generation of primary neurospheres from the dorsal lateral ganglionic eminence of late embryonic mice
Source: F1000Res. 2020 Mar 11;8:1983. Originally published 2019 Nov 25. [Version 2] doi: 10.12688/f1000research.21208.2 (PMC7101018; doi:10.12688/f1000research.21208.2)
Supplement: Peer review history

# Peer review: Reviewer response for version 2

**DOI:** 10.5256/f1000research.25140.r61177

**Published:** 2020-03-25

**Authors:** Oleksii Shandra

**Affiliations:** 1 Fralin Biomedical Research Institute, Virginia Tech Carilion, Roanoke, VA, USA; 2 Virginia Polytechnic Institute & State University, Blacksburg, VA, USA

All the comments made to the previous version have been addressed.

 Please, consider correcting a few minor typos:

**Data Acquisition and Statistics:**

 Typo: 

1. “steromicroscope” should be “stereomicroscope”.
2. “focus on the desire region” should be “focus on the desired desired region”.
3. “Data is” should be “data are”.

**Section 3. Primary neurosphere culture:**

 Repetition in the “NOTE”: “will need”, “will be needed”.

I confirm that I have read this submission and believe that I have an appropriate level of expertise to confirm that it is of an acceptable scientific standard.

# Peer review: Reviewer response for version 1

**DOI:** 10.5256/f1000research.23347.r57030

**Published:** 2019-12-24

**Authors:** Oleksii Shandra

**Affiliations:** 1 Fralin Biomedical Research Institute, Virginia Tech Carilion, Roanoke, VA, USA; 2 Virginia Polytechnic Institute & State University, Blacksburg, VA, USA

This method article written by Dr. Blackwood is aimed to provide a simple and reproducible protocol generating neurospheres from lateral ganglionic eminence in late embryonic mice and demonstrate its applicability for further immunocytochemical assays. Several aspects and protocol steps, however, require clarification:

1. This protocol includes multiple sections with crucial steps. One suggestion that could make the protocol even greater for educational purposes is providing a brief paragraph to sections 1, 2 and 3 (prior to individual steps) that will explain what is important in this section and list all the necessary reagents and tools that will be used. This will help anyone who is using this protocol for the first time to prepare all required items for this protocol section easier rather than finding the specific reagents and volume from each individual step.
2. Table 1 details equipment, reagents and catalogue numbers; however, the digital camera used for acquiring the images is not listed. The model of stereo stereomicroscope (step 2.5) should be added to table 1, as well as the optimum magnification required or used in this protocol. The type and model of humidified incubator in step 3.19 would also be helpful for reproducibility.
3. Section 3 called “primary neurosphere culture” is the main section explaining the method of neurosphere generation. In other publications, e.g. Raponi et al. (2007) ^1^, a filtration using a 40um-mesh-nylon cell strainer to dissociate cells after triturating. It may be valuable to add a note after trituration step that at this point similar step could be done if the purpose is to obtain uniform suspension from tissue.
4. The “Data acquisition and statistics” section requires more details. Specifically, the additional valuable information could be addressing the distance from the culture plate, whether this is an important factor for further size measurements and if so what was the distance between the camera and the well. What was the zoom factor used here?
5. The section “Primary neurospheres at 7 days *in vitro *can be used for immunocytochemistry” is aimed to demonstrate applicability of this protocol. The GFAP/DAPI is used as an example. While it is stated in the “Introduction” that neurospheres have been used to study molecular mechanisms controlling differentiation of both neural and glial cells, it is not very clear why GFAP was used for this example. Maybe author could clarify or explain why more specific markers were not used here instead of GFAP. From the Fig. 4 it looks as only 1 cell within the neurosphere was GFAP+, hence I believe using a more specific antibody could produce more representative result. Several references provided to this review detail markers that could also be used.
6. Lastly, I believe another interesting question that could be potentially valuable here is the step explaining the reader, which factors or characteristics of the neurosphere help to determine that the neurosphere is mature.

 

 In summary, after addressing these comments, I believe this method article is a good protocol that can be used as a reference and educational tool for both researchers that are familiar with similar techniques and those who approach it for the first time.

I confirm that I have read this submission and believe that I have an appropriate level of expertise to confirm that it is of an acceptable scientific standard, however I have significant reservations, as outlined above.

## References

[1] : S100B expression defines a state in which GFAP-expressing cells lose their neural stem cell potential and acquire a more mature developmental stage. *Glia*.2007;55(2) : 10.1002/glia.20445 165-77 10.1002/glia.20445 17078026PMC2739421

# Response

**Published:** 2020-03-08

**Authors:** Christopher Blackwood

**Affiliations:** Cornell University, USA

**Comment 2.1:**This protocol includes multiple sections with crucial steps. One suggestion that could make the protocol even greater for educational purposes is providing a brief paragraph to sections 1, 2 and 3 (prior to individual steps) that will explain what is important in this section and list all the necessary reagents and tools that will be used. This will help anyone who is using this protocol for the first time to prepare all required items for this protocol section easier rather than finding the specific reagents and volume from each individual step.

**Response 2.1:  **Thank you for your comments. We have revised accordingly and provided a brief paragraph to section 1, 2, and 3 prior to the individual steps.

1. Section 1: **Set-up prior to tissue dissection**

NOTE: *Breeding and euthanasia of all animals should be performed in accordance with an institutionally approved animal care and use protocol.* *Sterilize all surgical instruments packed in aluminum foil in an autoclave at 121°C (15 psi) for 30mins. This includes a scissors, forceps, and razor blades.  Before starting all premade solutions should be warmed to 37°C.*

1. Section 2: **Tissue dissection**

NOTE: *Make freshly prepared 70% ethanol spray prepared. Have a petri dish (100 mm) prepared with* *ice-cold Hank’s buffer kept on ice that will be used for embryos after dissection. Afterwards, additional petri dishes will be needed to place in each dissected brain (35 mm)*

Section 3: **Primary neurosphere culture**

*NOTE: Before starting warmed to 37°C the following solutions: trypsin/EDTA serum media, Hank’s-low, and Hank’s-high. In this section you will need the 18-gauge, 21-gauge, 23-gauge needle will be needed for trituration steps. Trituration should be performed gently and slowly to avoid killing cells. Hemocytometer will be needed to count cells.*

**Comment 2.2 **Table 1 details equipment, reagents and catalogue numbers; however, the digital camera used for acquiring the images is not listed. The model of stereo stereomicroscope (step 2.5) should be added to table 1, as well as the optimum magnification required or used in this protocol. The type and model of humidified incubator in step 3.19 would also be helpful for reproducibility.

**Response 2.2**. We have revised the Table 1 to add the Camera and Humidifier.   We also included information for the optimum magnification to Data acquisition and statistics section. 

“Images were taken with a Canon EOS Rebel XS camera. (Canon USA; Melville, NY). The optimum magnification is approximately 5x with 3888 x 2592 dimensions. Camera was connected to the trinocular port of the stereomicroscope (Carl Zeiss Stemi 305; White Plains, NY) using Mount Adaptor EF-EOS (6098B007AA; Canon; Melville, NY). 

**Comment 2.3 **Section 3 called “primary neurosphere culture” is the main section explaining the method of neurosphere generation. In other publications, e.g. Raponi et al. (2007) ^1^, a filtration using a 40um-mesh-nylon cell strainer to dissociate cells after triturating. It may be valuable to add a note after trituration step that at this point similar step could be done if the purpose is to obtain uniform suspension from tissue.  

**Response 2.3**. Thank you have revised the “primary neurosphere culture” section accordingly. “After trituration, if a uniform suspension has not been achieved an alternative method used in previous protocols are strainers ^14^.”

**Comment 2.4 **The “Data acquisition and statistics” section requires more details. Specifically, the additional valuable information could be addressing the distance from the culture plate, whether this is an important factor for further size measurements and if so what was the distance between the camera and the well. 

**Response 2.4: **We have revised the Data acquisition and statistics” section according to your recommendation. The scale bar should be used to advise the size of the neurosphere. Additionally, we have included information about the working distance to the “Data acquisition and statistics” section.

“The working distance was defined as the amount of room required between the top of the neurosphere and the bottom of the objective lens in order for the image to be in focus. The stereomicroscope was used at a working distance of ~110 mm. Due to the variation in neurosphere size, 110 mm should be adjusted to focus on the desire region of the neurosphere to provide optimal focus.”

**Comment 2.5: **The section “Primary neurospheres at 7 days *in vitro* can be used for immunocytochemistry” is aimed to demonstrate applicability of this protocol. The GFAP/DAPI is used as an example. While it is stated in the “Introduction” that neurospheres have been used to study molecular mechanisms controlling differentiation of both neural and glial cells, it is not very clear why GFAP was used for this example. Maybe author could clarify or explain why more specific markers were not used here instead of GFAP. From the Fig. 4 it looks as only 1 cell within the neurosphere was GFAP+, hence I believe using a more specific antibody could produce more representative result. Several references provided to this review detail markers that could also be used.

**Response 2.5:  **To provide more evidence that this protocol can be used to stain neurospheres, we have included an additional picture to the panel (Figure 4C). Larger neurospheres will contain more GFAP-expressing neurons that can be labeled.  Furthermore, we have reference our previous published report that demonstrated other immunocytochemistry using other antibodies. 

**Comment 2.6: **Lastly, I believe another interesting question that could be potentially valuable here is the step explaining the reader, which factors or characteristics of the neurosphere help to determine that the neurosphere is mature. 

**Response 2.6: **Thank you for the recommendation. We have revised the manuscript to expand this point in our discussion. 

“A sign of bacteria contamination is reduced visibility of the media. Another marker of an unhealthy culture is a large number of differentiated neurons surrounding neurospheres. Indicators of differentiation are the large presence of axons and dendrites in your cultures.This can be caused by depletion of growth factors. If this is the case, we recommend that you increase the concentration of EGF. Another cause of differentiation is too many cells in your prep. This leads to over crowdedness. We recommend splitting the culture to a lower density and decreasing the number of neurons that are plated per 48 well.”

# Peer review: Reviewer response for version 1

**DOI:** 10.5256/f1000research.23347.r57031

**Published:** 2019-12-19

**Authors:** M.L. Shawn Bates

**Affiliations:** 1 Division of Stress Neurobiology, Department of Anesthesiology and Critical Care, Abramson Research Center, Children's Hospital of Philadelphia, Philadelphia, PA, USA

This manuscript describes a protocol that can be used to generate primary neurospheres from the tissue surrounding the lateral ventricle in mice in the late embryonic period. The method is a relatively simple technique that will provide investigators with viable neurospheres that can be used in further studies.

 The protocol described here is important, and will likely be useful for many researchers who utilize neural stem cells. However, I have some constructive criticism and points that may require clarification, see below:

1. How long does it usually take to generate neurospheres? Is the fact that this protocol takes 7 days the rationale for developing it?
2. Is there a benefit to generating different sizes of neurospheres?
3. Why only use anti-GFAP (in essence, my question is why only visualize glia)?
4. Under the “Data acquisition and statistics subsection”:
  - The field of view represent **s** a length of...
  - Bonferroni's multiple comparis **o**n post hoc test…
  - A total of 5 individual animal **s** were analyzed…
5. The significance of the protocol is somewhat understated. I would add language to the Discussion to highlight the novelty of this approach. I would also include more suggestions for potential ways that others can use the protocol.

I confirm that I have read this submission and believe that I have an appropriate level of expertise to confirm that it is of an acceptable scientific standard.
